# Influenced from the start: anchoring bias in time trade-off valuations

**DOI:** 10.1007/s11136-016-1266-x

**Published:** 2016-03-26

**Authors:** Liv Ariane Augestad, Knut Stavem, Ivar Sønbø Kristiansen, Carl Haakon Samuelsen, Kim Rand-Hendriksen

**Affiliations:** 1 Department of Health Management and Health Economics, Faculty of Medicine, University of Oslo, Postboks 1089, Blindern, 0317 Oslo, Norway; 2 Health Services Research Center, Akershus University Hospital, Akershus, Norway; 3 Department of Pulmonary Medicine, Akershus University Hospital, Lørenskog, Norway; 4 Medical Faculty, Faculty Division, Akershus University Hospital, University of Oslo, Lørenskog, Norway

**Keywords:** Time trade-off, Health state valuation, Starting point, Anchoring bias, EQ-5D

## Abstract

**Purpose:**

The de facto standard method for valuing EQ-5D health states is the time trade-off (TTO), an iterative choice procedure. The TTO requires a starting point (SP), an initial offer of time in full health which is compared to a fixed offer of time in impaired health. From the SP, the time in full health is manipulated until preferential indifference. The SP is arbitrary, but may influence respondents, an effect known as *anchoring* bias. The aim of the study was to explore the potential anchoring effect and its magnitude in TTO experiments.

**Methods:**

A total of 1249 respondents valued 8 EQ-5D health states in a Web study. We used the lead time TTO (LT-TTO) which allows eliciting negative and positive values with a uniform method. Respondents were randomized to 11 different SPs. Anchoring bias was assessed using OLS regression with SP as the independent variable. In a secondary experiment, we compared two different SPs in the UK EQ-5D valuation study TTO protocol.

**Results:**

A 1-year increase in the SP, corresponding to an increase in TTO value of 0.1, resulted in 0.02 higher recorded LT-TTO value. SP had little impact on the relative distance and ordering of the eight health states. Results were similar to the secondary experiment.

**Conclusion:**

The anchoring effect may bias TTO values. In this Web-based valuation study, the observed anchoring effect was substantial. Further studies are needed to determine whether the effect is present in face-to-face experiments.

## Introduction

Health gain estimates in terms of quality-adjusted life years (QALYs) play an important role in healthcare resources allocation. EQ-5D is the most frequently used instrument for this purpose [1], and numerous national EQ-5D value algorithms have been developed. Since the seminal UK “Measuring and Valuing Health” EQ-5D valuation study in 1993, the time trade-off (TTO) method has been the de facto standard method for eliciting EQ-5D health state values [2]. The TTO method aims to elicit the point of indifference between a certain length of time in impaired health and a shorter time in full health. Setting the value of full health to 1 allows us to estimate the value of the impaired health state on a scale appropriate for QALY calculation. The point of indifference is identified through a series of discrete choices in which a fixed number of years in impaired health is compared to a variable number of years in full health until the respondent states preferential indifference. Several different *search procedures*—systems for varying the length of the life in full health—exist to reach the offer which represents the preferential equilibrium. Common to all such series of iterative choices is that they require an initial offer—*the starting point*. The rest of the search procedure consists of a pathway of subsequent offers, depending on the choices of the respondent, which we will refer to as *the routing*.

In the TTO variant usually employed in EQ-5D valuation studies, the task starts with a control question comparing 10 years in the impaired state to 10 years in full health. If the respondent prefers impaired health to full health or is indifferent, the task is explained again. Following this, the starting point of the task proper is 0 years in full health (equivalent to “immediate death”). For most applications of the TTO, the method for eliciting worse-than-death (WTD) values has been different from the method for eliciting better-than-death (BTD) values, and starting at 0 is a practical way of determining which of the two types of elicitation methods to proceed with [3, 4]. To our knowledge, neither theory nor literature offers an *a priory* correct starting point. The choice is therefore arbitrary in essence.

As long as the assumption of procedural invariance holds, the choice of starting point is of no consequence. However, if the choice of starting point influences respondents, the resulting TTO values will be influenced by a theoretically irrelevant factor [5, 6]. There is a considerable amount of evidence in the behavioral sciences that theoretically irrelevant factors, such as the starting point, may substantially influence judgments [7]. In this literature, bias associated with the starting point is usually referred to as “the anchoring effect” and is conceived as an inadequate adjustment from an initial starting point. Anchoring bias has been documented in many different areas of human judgment [8], including valuing health using the person trade-off method, [9], willingness to pay, and contingent valuation [10, 11]. Previous studies suggest that low familiarity, low relevance, and low personal involvement are factors that influence the magnitude of the anchoring effect [12]. In the context of valuing health states for national EQ-5D tariffs, we are not only dealing with a hypothetical trading situation; respondents value health states which they may never have experienced, using a highly unfamiliar “currency”—trading lifetime. A recently published paper on expected biases in iterative health state valuation protocols lists anchoring bias as one of the several important factors to consider and test empirically [13].

The research on search procedures using TTO has so far been limited to direct comparison of certain specific search procedures, concluding that values vary systematically depending on whether they are elicited with the “ping-pong”, top-down incremental or bottom-up incremental methods [14, 15]. Some of these observed discrepancies may be caused by using different starting points, but it is difficult to untangle the effect from the rest of the routing procedure, for instance, related to whether the subsequent offers are framed as gains or losses. Furthermore, the effect of the starting point could be different depending on the rest of the routing procedure.

The MVH protocol involves different elicitation methods for BTD and WTD values, which could make it difficult to isolate a potential anchoring effect. Furthermore, the protocol employs a “ping-pong” routing (partial bisection): A method in which the life in full health is traded back and forth between high and low values to close in on the respondents’ point of indifference. The ping-pong routing has an extended number of possible variations which could influence respondents in different ways, and which would be difficult to isolate from the anchoring effect. We therefore chose to focus on a variant of the TTO called the lead time TTO (LT-TTO), in which the same elicitation method is used for BTD and WTD values, and an incremental routing procedure, simply going one step up or down from the starting point, depending on the respondent’s answers. In the LT-TTO, a fixed period of “lead time” in full health is added prior to both the life in impaired health and the life in full health. Negative values are expressed by trading away lead time [16–18].

In this study, we investigated whether respondents were influenced by anchoring in TTO tasks, by using different starting points for the iterative choice procedure. To isolate the potential anchoring effect from other procedural effects, we used an incremental routing in an LT-TTO exercise. As a secondary analysis, we also included a study arm in which we applied the classical MVH TTO and routing, using two different starting points.

## Methods

### Study population

The study population was drawn from a Web panel organized by Synovate, a global market research company that has since been bought up by Ipsos. Respondents were primarily recruited to the panel through routinely asking participants in random telephone and postal studies whether they would be interested in participation. Approximately 40,000 individuals were listed in the Web panel at the time of the recruitment to our study. Our respondents were sampled from the panel to represent the Norwegian population aged 18–85 years and were invited by e-mail (*n* = 2234). Respondents were given a lottery-based incentive, with a draw of three universal gift cards, one of NOK 10,000 and two of NOK 5000 (approx 1700 and 850 USD, respectively).

### EQ-5D

Participants valued health states described by the EuroQol (EQ-5D-3L) descriptive system. The EQ-5D-3L categorizes health along five dimensions: mobility, self-care, usual activities, pain/discomfort, and anxiety/depression, each specified at three levels, corresponding to (1) no problems, (2) moderate problems, or (3) extreme problems. This allows description of 243 unique health states that are identified with a five-digit index ranging from 11111 for full health to 33333 for the worst possible health state [19]. The eight EQ-5D health states used in our experiments (11211, 11312, 22222, 11113, 32211, 23232, 32223, and 33333) were selected to cover a wide range of severities, based on mean values from previous valuation studies, while covering a variety of different types and levels of impairments. In order to evenly distribute potential influences or learning effects, the health states were presented to the respondents in a randomized order [20].

### Main experiment

In the main experiment, respondents compared two lives which both included 10 years of lead time in full health: In *Life A*, the lead time was followed by a variable number, from 0 to 10, of years in full health, resulting in a total Life A ranging from 10 to 20 years of full health. Respondents were randomized into 11 different staring points, referred to as starting point group 0–10, indicating the length of Life A in the first choice task. The initial offer in starting point group 0 was 10 years of full health, 11 years in group 1, 12 years in group 2, and 20 years in group 10. *Life B* always presented as 10 years in full health (lead time), followed by 10 years in the impaired target health state.

Life B was fixed throughout the experiment. From the respective starting points, the length of Life A was altered sequentially, depending on whether the respondent stated a preference for either Life A (next offer would be a shorter Life A) or Life B (next offer would be a longer Life A). For each offer of Life A, the respondents could state indifference between Life A and Life B, in which case the corresponding TTO value was recorded, and the respondent would continue valuing the next health state. At preference reversals, such as if a respondent preferred Life A at 12 years and subsequently opted for Life B at 11 years, the length of Life A would be altered by half a year. If the respondent could not arrive at preference equivalence using half-year increments, the value between the two options of half-year increments for which the preference reversal occurred was interpolated to a quarter of a year.

The respondent remained in the same starting point group for all experiments, i.e., if the first offer in Life A was 13 years of full health (starting point group 3), all eight health states would be valued using 13 years as a starting point. Establishing the length of Life A (*x*) at the (interpolated) point of indifference between Life A and Life B, the LT-TTO value *U* was calculated simply by subtracting the lead time of both lives and applying the standard formula for TTO values:

$$U_{i} = \frac{x - 10}{20 - 10}$$

**Fig. 1:**
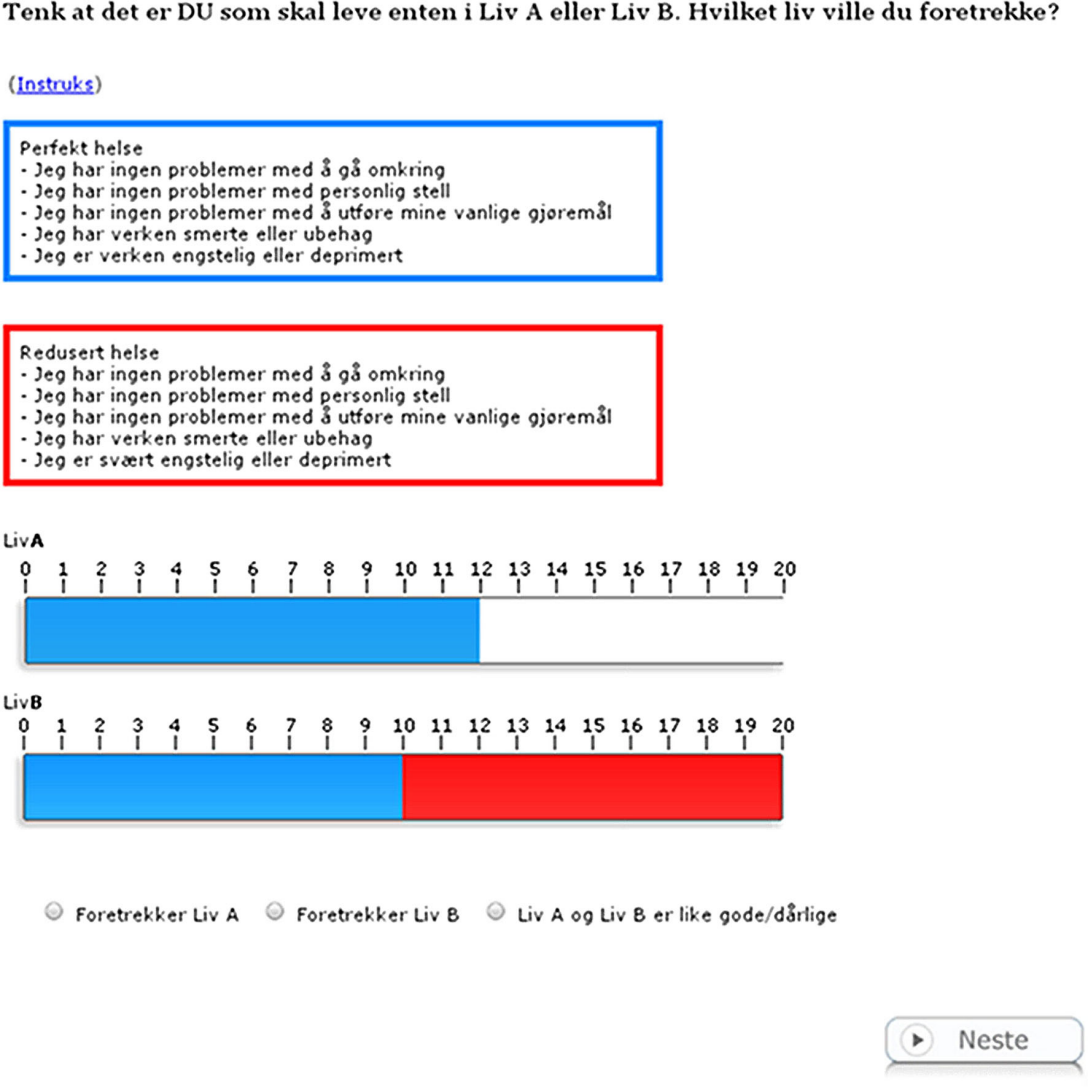
*Screen capture* of the LT-TTO experiment. The *upper rectangle* describes full health using the EQ-5D dimensions, while the text in the *rectangle* *underneath* describes one of the eight impaired EQ-5D health states used in this study. The text above translates into English “Imagine yourself in either Life A or Life B. Which one would you prefer?” In this case, Life A is at the starting point for LT-TTO group 2

In cases where the length of Life A is less than the lead time of 10 years at the point of indifference, the value for that particular health state is negative. This specification of the LT-TTO task, i.e., including 10 years of lead time, means that the lowest possible value a health state may receive is −1. The starting point of each group reflects a specific TTO value. Using 13 years as the starting point for Life A reflects a starting point LT-TTO value of 0.3. Figure 1 shows a screen capture of the main experiment.

### Secondary experiment

Respondents were randomized into two starting point groups (TTO and TTO + 5). The TTO group was administered a routing identical to the one used in the UK TTO valuation study [2, 21]. The UK and the most subsequent TTO protocols start off asking the respondent to state a preference between one life in the target state and one in full health when both are the same length. This question is included primarily to make sure the respondent is aware that the target state is impaired. If respondents state a preference for the impaired state, the interviewer is instructed to go through the task again. This first question does not involve trading of time, and we conceptualize it primarily as a control question. Subsequent to the control question, the initial offer was 0 years of full health (immediate death) compared to 10 years of target state. If the respondent considered the state to be BTD, the second offer was 5 years of full health, followed by death. From there, an iterative routing with 1-year increment was used. For WTD states, the second offer was 5 years in the target state, followed by 5 years full health, followed by death. This was compared to 0 years of full health (immediate death).

The group with the altered TTO version (TTO + 5) was given a starting point corresponding to the BTD part of the UK study protocol, with an initial comparison of 5 years in full health to 10 years in the target state. If the respondents exhausted the BTD scale by indicating that they preferred Life A, they were shifted to the WTD valuation procedure. In the TTO group and the TTO + 5 group, negative values were transformed to a 0 to −1 scale using the transformation method applied in the original UK valuation study. *t* is the number of years in full health in the combined life:

$$\begin{aligned} u_{i} & = \frac{ - t}{(10 - t)} \\ u_{i}^{\prime } & = \frac{u}{1 - u} = \frac{ - t}{10} \\ \end{aligned}$$

Respondents either participated in the main experiment or in the secondary experiment.

### Statistical analysis and exclusions

To control for response profiles that conveyed incomprehension of the TTO valuation task, we performed all analyses using two different inclusion regimes: one inclusive and one strict. In the strict regime, respondents who : (1) rated all eight health states as equal, (2) valued all health states as WTD or equal to death, or (3) rated the best state (11211) as worse than the worst state (33333) were excluded. In the inclusive regime, all respondents were included in analyses. The strict regime was used as the base case for the discussion.

We calculated the mean value for each of the EQ-5D health states by starting point group and for the TTO and TTO + 5 groups.

We used a multiple linear regression to investigate the relationship between the LT-TTO values and the 11 starting points. The regression used the LT-TTO values as the dependent variable, and age in years, sex (dummy variable indicating female), education (dummy variables representing 11–13 years and >13 years of education), and the TTO values corresponding to starting points as explanatory variables. To adjust for within-respondent correlation of responses across the eight health states, we included a random intercept for each individual. Using the same dependent and explanatory variables, we performed a robust regression as a sensitivity analysis.

Since anchoring could vary by health state, we also performed multiple regression analyses separately for each of the eight health states. We used the same predictors as in the previously listed analysis, except that the intercept was fixed, since there was only one observation per individual.

Some respondents may have been exhausted or not very engaged in the valuation task and may have stated indifference early to finish the task more quickly. This could lead to overestimating the anchoring effect. We therefore performed a sensitivity analysis in which we repeated the regression analysis described above, but excluding all responses that were a result of stating indifference at the first step.

### Secondary experiment

In order to compare TTO and TTO + 5, we used multiple regression with the elicited TTO value as the dependent variable, and a dummy variable representing TTO + 5 as the predictor of interest. In addition to this dummy variable, we included age in years, sex (dummy variable indicating female), and education (dummy variables representing 11–13 years and >13 years of education) as covariates. We performed this analysis separately for each health state and across all eight health states with random intercept at the level of individual respondents.

## Results

### Sample description

The demographic profile differed from the Norwegian population: Individuals with high education were overrepresented, and the main experiment survey included more females than the general population (Table 1). Table 2 describes the number of exclusions, mean values, and standard deviations for the all the respondent groups. Respondents with lower starting points triggered the inclusion criteria more often than respondents with higher starting points. The randomization process was designed so that each participant had about a 9 % chance of being in either of the starting point groups. This method left the starting point group 1 with notably fewer respondents than the other groups.

**Table 1:** Demographics

	Norway %	Strict *n* (%)			Inclusive *n* (%)		
		LT-TTO	Classic TTO	TTO-5	LT-TTO	Classic TTO	TTO-5
*n*		411	218	396	484	328	437
Age							
18–29	20	86 (20.9)	47 (21.6)	60 (15.2)	92 (19.0)	51 (15.5)	61 (14.0)
30–39	17.8	78 (19.0)	50 (22.9)	74 (18.7)	90 (18.6)	61 (18.6)	81 (18.5)
40–49	18.7	83 (20.2)	47 (21.6)	84 (21.2)	95 (19.6)	62 (18.9)	91 (20.8)
50–59	16.3	86 (20.9)	42 (19.3)	85 (21.5)	108 (22.3)	75 (22.9)	96 (22.0)
60–69	13.7	62 (15.1)	27 (12.4)	69 (17.4)	81 (16.7)	64 (19.5)	83 (19.0)
70–79	7.7	14 (3.4)	5 (2.39)	14 (3.5)	16 (3.3)	12 (3.7)	15 (3.4)
80+	5.8	2 (0.5)	–	–	2 (0.4)	3 (0.9)	–
Missing	–	–	–	10 (2.5)	–	–	10 (2.3)
Sex							
Male	49.6	183 (44.5)	107 (49.1)	196 (49.5)	217 (44.8)	153 (46.6)	217 (49.7)
Female	50.4	228 (55.5)	111 (50.9)	190 (48.0)	267 (55.2)	175 (53.4)	210 (48.1)
Missing	–	–	–	10 (2.5)	–	–	10 (2.3)
Education (years)							
8–10	29.8	27 (6.6)	13 (6.0)	23 (5.8)	35 (7.2)	26 (7.9)	29 (6.6)
11–13	42.9	132 (32.1)	77 (3.3)	129 (32.6)	163 (33.7)	102 (31.1)	147 (33.6)
>13	27.3	252 (61.3)	128 (58.7)	234 (59.1)	286 (59.1)	200 (61.0)	251 (57.4)
Missing	–	–	–	10 (2.5)	–	–	10 (2.3)

**Table 2:** Group characteristics and mean TTO values

Group		Inclusive		Strict		Exclusions	
Starting point TTO value		*n*	Mean (SD)	*n*	Mean (SD)	*n* (%)	Mean (SD)
LT-TTO	0–>1	484	0.16 (0.58)	411	0.24 (0.53)	73 (15)	−0.27 (0.63)
Group 0	0	29	−0.08 (0.61)	21	0.08 (0.52)	8 (28)	−0.47 (0.64)
Group 1	0.1	47	−0.12 (0.64)	34	0.09 (0.58)	13 (28)	−0.65 (0.43)
Group 2	0.2	45	0.13 (0.48)	38	0.2 (0.45)	7 (16)	−0.27 (0.45)
Group 3	0.3	43	0.13 (0.53)	37	0.19 (0.5)	6 (14)	−0.23 (0.52)
Group 4	0.4	49	0.14 (0.60)	38	0.25 (0.54)	11 (22)	−0.20 (0.64)
Group 5	0.5	54	0.18 (0.53)	48	0.27 (0.46)	6 (11)	−0.59 (0.43)
Group 6	0.6	42	0.24 (0.53)	34	0.29 (0.49)	8 (19)	0.00 (0.58)
Group 7	0.7	51	0.30 (0.54)	46	0.32 (0.51)	5 (10)	0.14 (0.68)
Group 8	0.8	33	0.27 (0.59)	28	0.3 (0.56)	5 (15)	0.09 (0.69)
Group 9	0.9	46	0.27 (0.58)	44	0.28 (0.58)	2 (4)	0.25 (0.48)
Group 10	1	45	0.24 (0.61)	43	0.26 (0.58)	2 (4)	−0.05 (0.99)
Classic TTO	0	328	−0.08 (0.72)	218	0.28 (0.56)	110 (33)	−0.80 (0.39)
TTO + 5	0.5	437	0.31 (0.54)	396	0.36 (0.48)	41 (9)	−0.17 (0.78)

### LT-TTO regression model

**Fig. 2:**
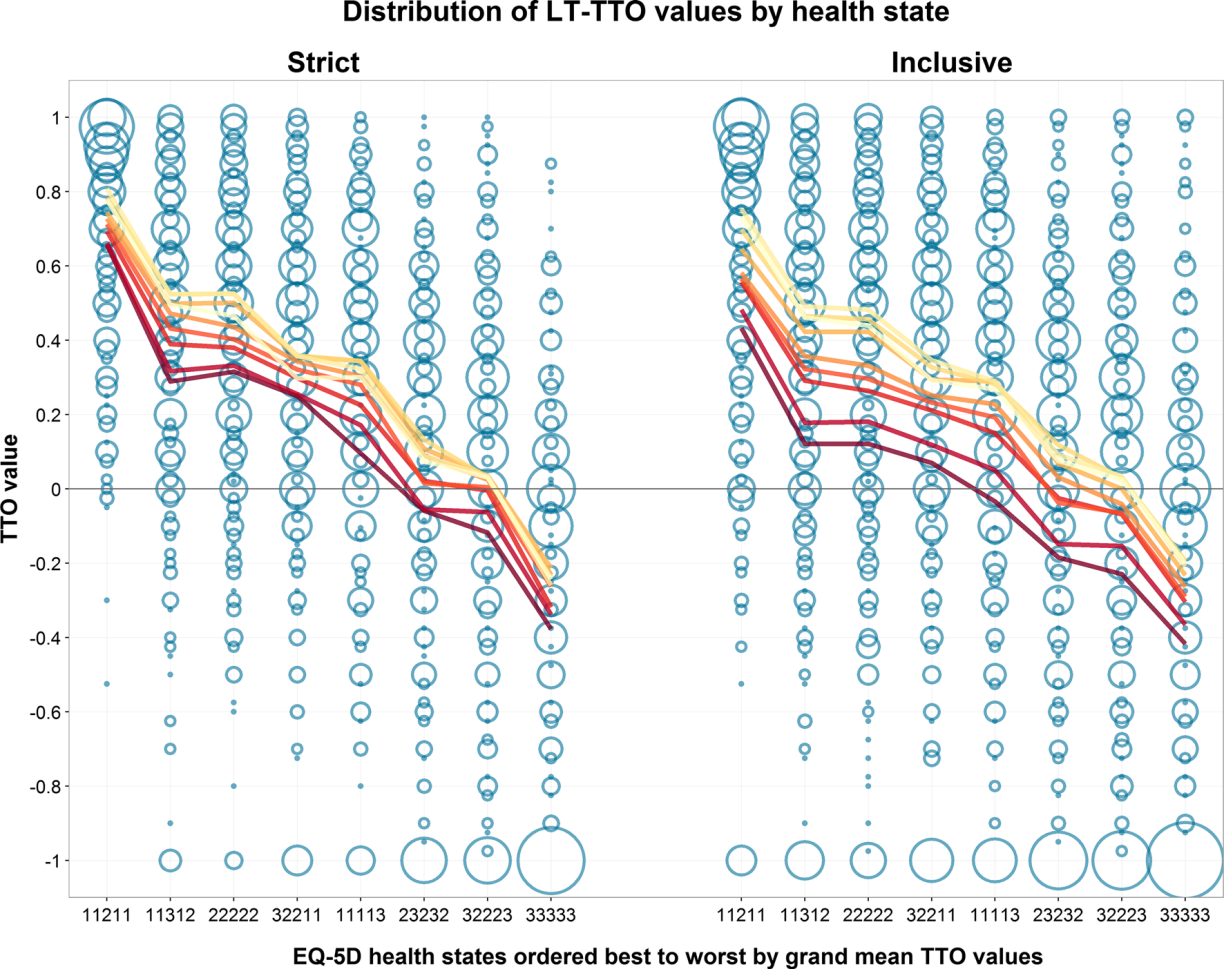
*Area of circles* proportional to number of respondents assigning specified value to the state in question. *Lines* represent mean values by three adjacent starting point group, such that the *darkest line* represent the lower starting points and the *lightest* the highest starting points

Inspection of mean values for each health state by starting point groups showed a persistent pattern of higher values with higher starting point group across the level of severity of health states (Fig. 2).

The regression analysis of the LT-TTO values (Table 3) indicated that an increase in the starting point of 1 year (0.1 on the TTO scale) resulted in a mean shift of the point of preferential indifference by 0.19 years, equivalent to an increase in TTO value of 0.019 (*p* < 0.001) in the strict setting, and 0.037 (*p* < 0.001) in the inclusive setting. The difference in mean values across all health states between the lowest and the highest starting point group was 0.19 (strict) and 0.37 (inclusive). Using robust regression, the coefficient for starting point value for the strict inclusion criteria was 0.025.

**Table 3:** Regression analyses by health state and across all health states—coefficient (*p* value)

EQ-5D health state	All^a^	11211	11312	22222	11113	32211	23232	32223	33333
*(a) Strict condition (after exclusions)*									
Starting point value	0.194 (<0.001)	0.140 (0.001)	0.261 (<0.001)	0.206 (0.001)	0.257 (<0.001)	*0.070 (0.333)*	0.232 (0.003)	0.197 (0.019)	0.189 (0.017)
Age	*−0.000 (0.402)*	*−0.000 (0.452)*	*−0.000 (0.648)*	*0.000 (0.857)*	*−0.000 (0.644)*	*−0.002 (0.150)*	*−0.003 (0.044)*	*0.001 (0.469)*	*−0.001 (0.344)*
Sex (1 = female, 0 = male)	*−0.005 (0.860)*	*0.005 (0.833)*	*0.056 (0.158)*	*−0.006 (0.872)*	*0.003 (0.930)*	*−0.033 (0.450)*	*−0.041 (0.400)*	*−0.017 (0.730)*	*−0.014 (0.764)*
Education 1 (11–13 years)	*−0.008 (0.911)*	*−0.045 (0.449)*	*−0.044 (0.615)*	*−0.057 (0.513)*	*−0.059 (0.537)*	*0.002 (0.981)*	*0.028 (0.792)*	*0.064 (0.565)*	*0.046 (0.659)*
Education 2 (>13 years)	*0.051 (0.450)*	*0.022 (0.692)*	*−0.005 (0.946)*	*0.023 (0.775)*	*−0.035 (0.694)*	*0.027 (0.761)*	*0.129 (0.196)*	*0.123 (0.240)*	*0.128 (0.196)*
Constant	*0.152 (0.113)*	0.684 (<0.001)	0.310 (0.007)	0.312 (0.006)	*0.190 (0.136)*	0.372 (0.003)	*0.006 (0.963)*	*−0.257 (0.082)*	−0.394 (0.004)
*(b) Inclusive condition: all participants*									
Starting point value	0.373 (<0.001)	0.395 (<0.001)	0.453 (<0.001)	0.430 (<0.001)	0.415 (<0.001)	0.295 (<0.001)	0.381 (<0.001)	0.339 (<0.001)	0.278 (<0.001)
Age	−0.002 (0.043)	−0.003 (0.009)	−0.003 (0.038)	*−0.002 (0.082)*	*−0.002 (0.094)*	−0.003 (0.029)	−0.003 (0.025)	*−0.000 (0.813)*	*−0.001 (0.455)*
Sex (1 = female, 0 = male)	−0.086 (0.021)	*−0.059 (0.135)*	*−0.026 (0.550)*	*−0.067 (0.134)*	*−0.103 (0.024)*	−0.111 (0.016)	−0.127 (0.008)	*−0.084 (0.089)*	*−0.107 (0.025)*
Education 1 (11–13 years)	*0.041 (0.596)*	*0.049 (0.551)*	*0.033 (0.720)*	*0.003 (0.972)*	*−0.006 (0.946)*	*0.078 (0.422)*	*0.075 (0.449)*	*0.065 (0.528)*	*0.032 (0.745)*
Education 2 (>13 years)	*0.107 (0.145)*	0.161 (0.041)	*0.097 (0.268)*	*0.103 (0.243)*	*0.026 (0.766)*	*0.112 (0.223)*	0.158 (0.095)	*0.125 (0.202)*	*0.077 (0.416)*
Constant	*0.062 (0.563)*	0.482 (<0.001)	0.198 (0.121)	0.201 (0.121)	*0.130 (0.321)*	0.205 (0.124)	*−0.085 (0.538)*	−0.267 (0.062)	−0.368 (0.008)

Statistically nonsignificant values (*p* ≥ 0.05) are italicized

^a^Regression with random intercept for each participant

**Fig. 3:**
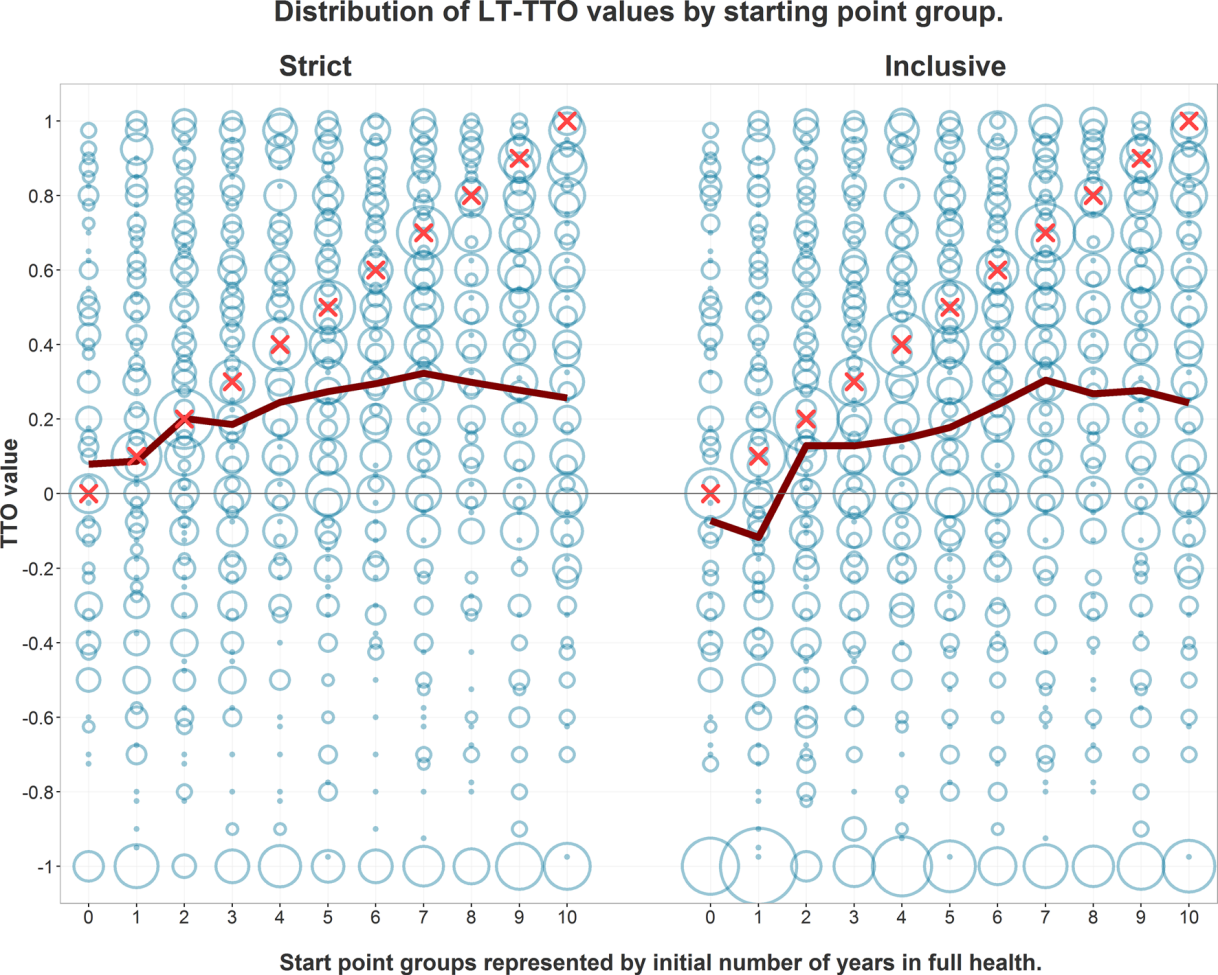
*Area of circles* is proportional to number of respondents in group assigning specified TTO value, across all eight health states. *Red crosses* represent value at the groups’ starting points. *Thick line* represents mean value across all eight health states. (Color figure online)

Stratifying the responses by the eight health states reveals that the starting point groups seem to agree on the relative distance between the health states (Fig. 3).

With strict inclusion criteria, the random effects regression model predicting TTO values across health states resulted in an estimated influence from the starting point of 0.194 (*p* < 0.001). The starting point was statistically significant for seven of the eight health states when analyzed separately (Table 3a). There was no clear pattern of increasing or decreasing coefficients with severity of the health states.

With all participants included in the analyses, the estimated influence from the starting point was substantially greater (0.373 on average) and statistically significant (*p* < 0.001) in all the state-specific models (Table 3b).

Leaving out responses where indifference was stated at the first step (i.e., the starting point) resulted in a slight decrease of 0 0.03 for the starting point coefficient in both the strict and inclusive setting (Table 4).

**Table 4:** Regression analyses excluding responses at starting point value, by health state and across all health states—coefficient (*p* value)

EQ-5D health state	All^a^	11211	11312	22222	11113	32211	23232	32223	33333
*(a) Strict condition (after exclusions)*									
Starting point value	0.161 (0.006)	0.140 (0.001)	0.261 (<0.001)	0.206 (0.001)	0.257 (<0.001)	*0.070 (0.333)*	0.232 (0.003)	0.197 (0.019)	0.189 (0.017)
Age	*−0.001 (0.246)*	*−0.000 (0.452)*	*−0.000 (0.648)*	*0.000 (0.857)*	*−0.000 (0.644)*	*−0.002 (0.150)*	−0.003 (0.044)	*0.001 (0.469)*	*−0.001 (0.344)*
Sex (1 = female, 0 = male)	*−0.018 (0.616)*	*0.005 (0.833)*	*0.056 (0.158)*	*−0.006 (0.872)*	*0.003 (0.930)*	*−0.033 (0.450)*	*−0.041 (0.400)*	*−0.017 (0.730)*	*−0.014 (0.764)*
Education 1 (11–13 years)	*0.043 (0.584)*	*−0.045 (0.449)*	*−0.044 (0.615)*	*−0.057 (0.513)*	*−0.059 (0.537)*	*0.002 (0.981)*	*0.028 (0.792)*	*0.064 (0.565)*	*0.046 (0.659)*
Education 2 (>13 years)	*0.114 (0.127)*	*0.022 (0.692)*	*−0.005 (0.946)*	*0.023 (0.775)*	*−0.035 (0.694)*	*0.027 (0.761)*	*0.129 (0.196)*	*0.123 (0.240)*	*0.128 (0.196)*
Constant	*0.118 (0.261)*	0.684 (<0.001)	0.310 (0.007)	0.312 (0.006)	*0.190 (0.136)*	0.372 (0.003)	*0.006 (0.963)*	*−0.257 (0.082)*	−0.394 (0.004)
*(b) Inclusive condition: all participants*									
Starting point value	0.343 (<0.001)	0.395 (<0.001)	0.453 (<0.001)	0.430 (<0.001)	0.415 (<0.001)	0.295 (<0.001)	0.381 (<0.001)	0.339 (<0.001)	0.278 (<0.001)
Age	−0.003 (0.014)	−0.003 (0.009)	−0.003 (0.038)	*−0.002 (0.082)*	*−0.002 (0.094)*	−0.003 (0.029)	−0.003 (0.025)	*−0.000 (0.813)*	*−0.001 (0.455)*
Sex (1 = female, 0 = male)	−0.098 (0.014)	*−0.059 (0.135)*	*−0.026 (0.550)*	*−0.067 (0.134)*	−0.103 (0.024)	−0.111 (0.016)	−0.127 (0.008)	*−0.084 (0.089)*	−0.107 (0.025)
Education 1 (11–13 years)	*0.070 (0.397)*	*0.049 (0.551)*	*0.033 (0.720)*	*0.003 (0.972)*	*−0.006 (0.946)*	*0.078 (0.422)*	*0.075 (0.449)*	*0.065 (0.528)*	*0.032 (0.745)*
Education 2 (>13 years)	*0.148 (0.060)*	0.161 (0.041)	*0.097 (0.268)*	*0.103 (0.243)*	*0.026 (0.766)*	*0.112 (0.223)*	*0.158 (0.095)*	*0.125 (0.202)*	*0.077 (0.416)*
Constant	*0.052 (0.644)*	0.482 (<0.001)	0.198 (0.121)	0.201 (0.121)	*0.130 (0.321)*	0.205 (0.124)	*−0.085 (0.538)*	−0.267 (0.062)	−0.368 (0.008)

Statistically nonsignificant values (*p* ≥ 0.05) are italicized

^a^Regression with random intercept for each participant

### Comparison of the TTO and TTO + 5

The regression analysis indicated that TTO + 5 resulted in 0.09 higher values than TTO (*p* < 0.001) (Table 5). The respondents who were administered TTO, were much more likely to trigger the exclusion criteria (110 out of the 328 respondents) than those who were administered the TTO + 5 (41 out of 437). Because many of the exclusions were related to giving all health states values below zero, the exclusions reduced the mean difference in elicited TTO values between the two groups substantially, from 0.40 in the inclusive setting to 0.09 in the strict setting.

**Table 5:** Regression analyses comparing classical TTO with TTO + 5, by health state and across all health states—coefficient (*p* value)

EQ-5D health state	All^a^	11211	11312	22222	11113	32211	23232	32223	33333
*(a) Strict condition (after exclusions)*									
TTO variant (1 = TTO5, 0 = TTO)	0.085 (0.002)	−0.040 (0.012)	*0.006 (0.858)*	*0.010 (0.770)*	0.101 (0.009)	*0.027 (0.480)*	0.168 (<0.001)	0.202 (<0.001)	0.206 (<0.001)
Age	*0.001 (0.273)*	*0.000 (0.389)*	*0.000 (0.622)*	0.002 (0.036)	*0.001 (0.466)*	*0.000 (0.492)*	*0.000 (0.575)*	*0.001 (0.307)*	*0.000 (0.575)*
Sex (1 = female, 0 = male)	*0.025 (0.342)*	*−0.001 (0.929)*	*0.013 (0.688)*	*0.041 (0.235)*	*−0.026 (0.470)*	*0.060 (0.101)*	*0.056 (0.141)*	*0.030 (0.420)*	*0.030 (0.407)*
Education 1 (11–13 years)	*0.026 (0.659)*	*0.029 (0.396)*	*−0.041 (0.576)*	*−0.015 (0.841)*	*0.152 (0.070)*	*0.018 (0.821)*	*0.064 (0.463)*	*0.046 (0.588)*	*−0.039 (0.627)*
Education 2 (>13 years)	*0.050 (0.378)*	*0.050 (0.119)*	*0.003 (0.955)*	*0.019 (0.797)*	*0.120 (0.131)*	*0.066 (0.401)*	*0.084 (0.306)*	*0.079 (0.331)*	*−0.018 (0.811)*
Constant	0.176 (0.024)	0.789 (<0.001)	0.494 (<0.001)	0.339 (<0.001)	*0.128 (0.236)*	0.274 (0.010)	*−0.092 (0.410)*	*−0.185 (0.093)*	−0.335 (0.001)
*(b) Inclusive condition: all participants*									
Starting point value	0.396 (<0.001)	0.368 (<0.001)	0.376 (<0.001)	0.376 (<0.001)	0.408 (<0.001)	0.375 (<0.001)	0.425 (<0.001)	0.444 (<0.001)	0.394 (<0.001)
Age	−0.006 (<0.001)	−0.010 (<0.001)	−0.008 (<0.001)	−0.006 (<0.001)	−0.006 (<0.001)	−0.007 (<0.001)	−0.005 (<0.001)	−0.004 (0.001)	−0.003 (0.015)
Sex (1 = female, 0 = male)	*−0.043 (0.228)*	*−0.075 (0.068)*	*−0.068 (0.117)*	*−0.045 (0.311)*	−0.088 (0.036)	*−0.018 (0.668)*	*−0.005 (0.883)*	*−0.027 (0.483)*	*−0.021 (0.547)*
Education 1 (11–13 years)	*0.043 (0.575)*	*0.052 (0.544)*	*0.010 (0.910)*	*0.025 (0.788)*	*0.122 (0.169)*	*0.019 (0.838)*	*0.078 (0.359)*	*0.050 (0.538)*	*−0.015 (0.833)*
Education 2 (>13 years)	*0.089 (0.211)*	*0.129 (0.111)*	*0.079 (0.353)*	*0.086 (0.325)*	*0.119 (0.152)*	*0.087 (0.315)*	*0.112 (0.160)*	*0.100 (0.192)*	*0.001 (0.983)*
Constant	*0.178 (0.087)*	0.791 (<0.001)	0.471 (<0.001)	0.326 (0.011)	*0.162 (0.180)*	0.286 (0.023)	*−0.100 (0.388)*	−0.170 (0.129)	−0.342 (<0.001)

Statistically nonsignificant values (*p* ≥ 0.05) are italicized

^a^Regression with random intercept for each participant

## Discussion

The results indicate that the values elicited using TTO was substantially influenced by the starting point of the task, supporting the anchoring hypothesis. The effect was observed both in the main experiment using LT-TTO and in the analysis that compared the TTO with the TTO + 5. This suggests that anchoring bias could be a problem in most TTO search procedures. The observed effects were substantial, with an estimated mean shift in values of 0.19 from the lowest to the highest starting point in the LT-TTO group.

Some of the exclusion criteria were related to assigning low health state values (all TTO values 0 or negative). Respondents with lower starting points were more likely to trigger exclusion criteria, which could be due in part to the anchoring effect. However, respondents with low starting points also had an increased tendency to assign higher values to the worst state than to the best, suggesting that the lower starting points may have made the task more difficult to perform.

Our manipulations were restricted to varying the starting point over TTO values corresponding from 0 to 1. Additional manipulations could increase the effect of the starting point: For example, different lengths of lead time, changing the increment size of tradable time, allowing negative starting points, increasing the visible length of the TTO bars, or offering health states with different durations would all potentially influence elicited values [22–24].

A way of limiting the effects of anchoring on estimated health state values could be to randomize the starting points in a way similar to this study. Unlike the fixed starting point approach, bias would then distribute over a range of health state values. The anchoring effects of high starting points could then to some extent be mitigated by the opposite effect of the low starting point for health states that have mid-range values. While a definite improvement over imposing a single starting point, values elicited using random starting points could still be shifted along the absolute value scale: If we assume that anchoring is a function of the distance between the initial offer and the theoretical unbiased (“true”) preference of the respondent, randomizing the starting point over a range of values should improve the validity of health state values on a relative scale. However, the impact on the absolute scale of interest would still remain unknown. We can surmise that varying the starting point from a maximum of 1 to some lower boundary will lead to lower mean values for health states close to full health, and that the net negative anchoring bias on mild states should be a function of how far down we allow the starting points to vary. A similar argument in the opposite direction can be made for severe health states, with the added complication that we have less information about the “true” values that should be expected for severe states, since our knowledge is limited to prior valuations, with their susceptibility to anchoring and various other issues.

### Limitations

TTO values are far from normally distributed, challenging the appropriateness of multiple linear regression. However, EQ-5D tariff modeling is intended to model observed mean values, and the methods used in valuation studies are based on multiple linear regression. Our methods thus reflect the influence of the potential anchoring effect, using a specific starting point in a valuation study setting. Further, keeping in mind that the sensitivity analysis using robust regression resulted in a larger coefficient for the anchoring effect, the discussion is based on the most conservative estimate of the anchoring effect.

The lack of face-to-face interaction in our Web-based study may increase the risk that respondents misunderstand the TTO valuation task or engage in satisficing behavior [25]. The causes for insufficient adjustment, assumed to be the driver of observed anchoring, are to a large extent unknown. As outlined in the introduction, anchoring is associated with low relevance and low personal involvement [12]. Kruger suggests that adjustment from an initial anchor requires cognitive effort, and that the anchoring effect is a result of trying to minimize cognitive effort [26]. Having an interviewer present in a face-to-face setting could potentially reduce the anchoring effect in several ways by raising the level of engagement and encouraging cognitive effort.

The comparison of the TTO and TTO + 5 groups should be made with caution. While there is little doubt that changing the routing procedure has substantial impact on elicited values, factors other than classical anchoring may be at play. For instance, respondents in the TTO group may react to the initial direct comparison to immediate death [27, 28].

It remains to be determined whether the observed anchoring effects are specific to TTO or whether the findings generalize to other iterative search-based indifference procedures, such as the standard gamble. The anchoring effect observed in the LT-TTO part of the study may be attenuated by fatigue effects, since varying the starting point influences the number of iterations required to reach specific values. However, the observed difference between classic TTO and TTO + 5 is less confounded with fatigue, but substantial. Furthermore, a previous study found limited evidence of fatigue effects in the US EQ-5D valuation study [20].

### Implications

The QALY framework requires health state values to be specified in absolute terms in relation to the reference points of death and full health. Since the anchoring effect shifts the value of all measured health states relative to the reference points, the valued health states’ absolute distance from these points becomes uncertain. The ordering and the relative distance between the eight EQ-5D health states varied little across both the starting point groups and the different TTO variants, suggesting that these properties are less sensitive to anchoring bias.

A basic problem with the anchoring effect is that we have no information on the individual’s unanchored value. Even if we observe anchoring at a group level, we can see no actual way of correcting the data once they are collected. It follows from the anchoring effect that the health state values are a function of an arbitrary starting point, which of course represents a threat to the validity of the values. It is therefore crucial to investigate whether the anchoring effect is present in other TTO settings, for instance the face-to-face setting, which remains the gold standard when performing EQ-5D valuation studies. If an effect similar to the one we observed is present, research aiming to gain knowledge as to how to reduce the anchoring effect would be important for designing future valuation studies. Another option would be to move away from sequential choice tasks which require a starting point, and use, for instance, discrete choice experiments.

## Conclusion

Values elicited using LT-TTO and TTO were influenced by anchoring from the essentially arbitrary starting point of the task, questioning the validity of the absolute values elicited with the TTO. Future research should focus on examining whether the anchoring effect is present in other TTO settings, especially in face-to-face interviews, since this remains the standard setting for eliciting TTO values. Research aimed at understanding what makes respondents susceptible to the anchoring effect could help design studies to reduce this bias in future valuation studies.
